# Stress and immunological response of heifers divergently ranked for residual feed intake following an adrenocorticotropic hormone challenge

**DOI:** 10.1186/s40104-017-0197-x

**Published:** 2017-08-08

**Authors:** A. K. Kelly, P. Lawrence, B. Earley, D. A. Kenny, M. McGee

**Affiliations:** 1 School of Agriculture and Food Science, College of Health and Agricultural Sciences, University College Dublin, Belfield, Dublin 4, Ireland; 2 Animal and Bioscience Research Department, Animal & Grassland Research and Innovation Centre, Teagasc, Dunsany, Co. Meath, Ireland; 3 Livestock Systems Research Department Teagasc, Animal & Grassland Research and Innovation Centre, Grange, Dunsany, Co. Meath, Ireland

**Keywords:** Beef cattle, Cortisol, Feed efficiency, Residual feed intake, Stress response

## Abstract

**Background:**

When an animal is exposed to a stressor, metabolic rate, energy consumption and utilisation increase primarily through activation of the hypothalamic-pituitary-adrenal (HPA) axis. Changes to partitioning of energy by an animal are likely to influence the efficiency with which it is utilised. Therefore, this study aimed to determine the physiological stress response to an exogenous adrenocorticotropic hormone (ACTH) challenge in beef heifers divergently ranked on phenotypic residual feed intake (RFI).

**Results:**

Data were collected on 34 Simmental weaning beef heifers the progeny of a well characterized and divergently bred RFI suckler beef herd. Residual feed intake was determined on each animal during the post-weaning stage over a 91-day feed intake measurement period during which they were individually offered adlibitum grass silage and 2 kg of concentrate per head once daily. The 12 highest [0.34 kg DM/d] and 12 lowest [−0.48 kg DM/d] ranking animals on RFI were selected for use in this study. For the physiological stress challenge heifers (mean age 605 ± 13 d; mean BW 518 ± 31.4 kg) were fitted aseptically with indwelling jugular catheters to facilitate intensive blood collection. The response of the adrenal cortex to a standardised dose of ACTH (1.98 IU/kg metabolic BW^0.75^) was examined. Serial blood samples were analysed for plasma cortisol, ACTH and haematology variables. Heifers differing in RFI did not differ (*P* = 0.59) in ACTH concentrations. Concentration of ACTH peaked (*P* < 0.001) in both RFI groups at 20 min post-ACTH administration, following which concentration declined to baseline levels by 150 min. Similarly, cortisol systemic profile peaked at 60 min and concentrations remained continuously elevated for 150 min. A RFI × time interaction was detected for cortisol concentrations (*P* = 0.06) with high RFI heifers had a greater cortisol response than Low RFI from 40 min to 150 min relative to ACTH administration. Cortisol response was positively associated with RFI status (*r* = 0.32; *P* < 0.01). No effect of RFI was evident for neutrophil, lymphocytes, monocyte, eosinophils and basophil count. Plasma red blood cell number (6.07 vs. 6.23; *P* = 0.02) and hematocrit percentage (23.2 vs. 24.5; *P* = 0.02) were greater for low than high RFI animals.

**Conclusions:**

Evidence is provided that feed efficiency is associated with HPA axis function and susceptibility to stress, and responsiveness of the HPA axis is likely to contribute to appreciable variation in the efficiency feed utilisation of cattle.

## Background

Selecting and propagating cattle genetics that are more efficient at converting feed into carcass gain is an important element of a profitable and sustainable livestock industry [1]. As a moderately heritable trait, independent of growth and body size, RFI is now a favoured measure of feed efficiency for livestock [2]. Independence of RFI from production also affords the opportunity to unravel the inherent variation in biological processes underpinning differences in inter-animal efficiency. Various biological processes have been identified as possible contributors to variation in feed efficiency [2–5], but no one process predominates.

Susceptibility to stress and biological differences in an animal’s stress response is proposed [6, 7] as probable drivers of variation in feed efficiency that warrant further investigation. Indeed, an association between stress susceptibility and energetic inefficiency is conceivable, given that one of the noted biological responses to a stressful stimuli is to increase metabolic rate, energy consumption and decrease immunity, through alterations in the functioning of neuro-endocrine-immune pathways [8, 9].

Stress response of animals is generally determined by measuring glucocorticoids and HPA axis activity [10]. The activity of the HPA axis can be stimulated by way of a physiological stress challenge (following exogenous administration of ACTH and/or corticotrophin-releasing hormone; CRH**)** and the stress response of the HPA can be assessed at the pituitary and adrenal levels. Knott et al. [6] reported that RFI and cortisol response were positively associated, in rams selected based on their response to an ACTH physiological stress challenge. In laying hens originating from a multi-generational divergent RFI selection line, Luiting et al. [11] reported that the inefficient hen lines had a greater cortisol response following an adrenal ACTH challenge compared to their efficient contemporaries. However, from a cattle perspective there is a dearth of published information on this topic. Therefore, the objective of this study was to determine whether endocrine and hematologic responses following an exogenous ACTH challenge differ between beef heifers divergent for phenotypic RFI.

## Methods

All procedures involving animals in this study were conducted under an experimental licence from the Irish Department of Health and Children in accordance with the cruelty to Animals Act 1876 and the European Communities (Amendment of Cruelty to Animals Act 1876) Regulation 2002 and 2005 (http://www.dohc.ie/other_health_issues/uaeosp).

### Animal management and determination of RFI

Performance and RFI data were collected on 34 Simmental weaning beef heifers which were the progeny of a well characterized and divergently bred RFI suckler beef herd established in Teagasc Grange, as described by [12–14]. During the post-weaning stage individual daily feed intake was recorded on heifers (mean initial BW = 299 ± 4 kg, mean initial age = 258 ± 27 d) over a 91-day period, following which RFI co-efficients were calculated for each animal. Heifers were housed in a slatted floor building with a Calan gate feeding system (American Calan Inc., Northwood, NH) and were given an adaptation period of 14 d to acclimatise to their diet and environment before recording individual intake began (RFI measurement period). Heifers were individually offered grass silage and 2 kg of concentrate once per day (at 0800 h). The concentrate offered contained 430 kg rolled barley, 430 kg beet pulp, 80 kg soya bean meal, 45 kg molasses and 15 kg minerals and vitamins per tonne. Grass silage allocation was based on approximately 1.1 times the previous day’s intake. The chemical composition and in vitro dry matter digestibility of the grass silage and concentrate offered is outlined in Table 1.

**Table 1:** Chemical composition of the dietary ingredients offered during the residual feed intake experimental period

	RFI experimental period	
Variable	Silage	Concentrate
Dry matter, g/kg^a^	315 ± 33.9	822 ± 0.7
Composition of DM, g/kg DM unless otherwise stated		
pH	3.9 ± 0.15	ND^b^
In vitro DMD	693 ± 47.3	726 ± 44.0
In vitro DOMD^c^	625 ± 37.1	676 ± 44.6
OMD^d^	677 ± 47.5	726 ± 46.9
Ash	75 ± 11.7	73 ± 1.6
Crude protein	121 ± 10.3	115 ± 11.6
NDF	532 ± 54.7	244 ± 31.4
Starch	ND	230 ± 56.3
ME, MJ/kg DM^e^	10.41 ± 0.45	11.56 ± 0.36
Fermentation characteristics^f^, g/kg		
Lactic acid	102 ± 49.8	ND
Acetic acid	33 ± 7.7	ND
Propionic acid	2.9 ± 0.66	ND
Butyric acid	4.5 ± 2.39	ND
Ethanol	13.4 ± 2.82	ND
Ammonia N, g/kg total N	76 ± 10.9	ND

^a^Corrected for loss of volatiles during oven drying

^b^ *ND* not determined

^c^Digestible OM in the total DM, measured in vitro

^d^OM digestibility, measured in vitro

^e^Estimated based on in vitro digestible OM in total DM (AFRC, 1993)

^f^Expressed as g/kg volatile corrected DM

Heifers were weighed (prior to feeding) on two consecutive days at the beginning and end of the period of RFI measurement and every 21 d during the experimental period. Average daily gain was computed as the coefficient of the linear regression of weight (kg) on time using the REG procedure of the Statistical Analysis Systems (SAS Institute Inc., Cary, NC). Mid-test metabolic body weight (MBW) was estimated from the intercept and slope of the regression line after fitting a linear regression through all metabolic body weight (BW^0.75^) observations. Feed conversion ratio (F:G**)** of each animal was computed as the ratio of daily DMI to ADG. Residual feed intake was calculated for each animal as the difference between actual DMI and expected DMI. Expected DMI was computed for each animal using a multiple regression model, regressing DMI on MBW and ADG. The model used was

$$ {\mathrm{Y}}_{\mathrm{j}}={\upbeta}_0+{\upbeta}_1{MLW}_{\mathrm{j}}+{\upbeta}_2{ADG}_{\mathrm{j}}+{\mathrm{e}}_{\mathrm{j}}, $$

Where Y_j_ is the average DMI of the jth animal, β_0_ is the partial regression intercept, β_1_ is the partial regression coefficient on MBW^0.75^, β_2_ is the partial regression coefficient on ADG, and e_j_ is the uncontrolled error of the jth animal. The coefficient of determination (*R* ^2^) from this model was equal to 0.66 (*P* < 0.001) and the model was subsequently used to predict DMI for each animal.

Heifers were turned out to pasture after the RFI experimental period ended. For the duration of the grazing season (7 mo), heifers were rotationally grazed as one group under a moderate stocking rate on predominately perennial ryegrass (*Lolium perenne* L.) pasture until housing at the end of October, when the grazing season ended [mean ADG of 0.80 kg/d (SEM = 0.03)]. For the physiological stress challenge experiment the 12 highest [mean (0.48 ± 0.08) kg/d]; High RFI and 12 lowest [mean − (0.50 ± 0.08) kg/d]; Low RFI ranking animals on post weaning RFI were selected.

### Jugular vein catheterisation

During the ACTH stress challenge the 24 heifers (mean age 605 ± 13 d; mean BW 516 ± 31.4 kg) were housed in a slatted-floor facility and were offered grass silage ad libitum*.* Start weight (*P =* 0.52) and age (*P =* 0.88) were not different between the high and low RFI groups. As part of standard husbandry and research management practices animals were accustomed to stockpersons and routine handling, approximately 14-day prior to the ACTH challenge. To facilitate intensive blood collection and minimize handling stress during blood sampling, heifers were fitted aseptically with indwelling jugular catheters on d −1. The procedure was using 12-gauge Anes spinal needles (Popper and Sons, Inc., New Hyde Park, NY) and polyvinyl tubing (approximately 1.47 mm i.d.; Ico Rally Corp., Palo Alto, CA; catalog No. SVL 105–18 CLR) attached to an 18-gauge needle at the blood collection end. After catheterization, catheter patency was maintained by flushing with 3.5% sodium citrate after each blood collection.

### ACTH challenge

The response of the adrenal cortex to a standardised dose of ACTH (1.98 IU/kg metabolic BW^0.75^) was examined. The dose chosen of exogenous ACTH and was previously established by our group [15] to be effective in adequately stimulating HPA responses, with resultant elevations in cortisol not sufficient to cause negative effects on animal health. Dexamethasone (20 μg/kg BW; Faulding Pharmaceuticals Plc, UK) was administered intramuscularly (i.m.) on d −1, 12.40 h prior to heifers undergoing ACTH challenge (Synacthen Ampoules, Novartis Pharmaceutical Ltd., UK) on the following morning (d 0). In cattle, DEX is rapidly absorbed and the elimination half-life of DEX has been reported by Toutain et al. to ranges from 291 to 335 min [16, 17]. Dexamethasone was administered to equalize systemic concentrations of cortisol [9] in animals across the RFI groups, in an effort to facilitate a more equitable examination of ACTH on cortisol release [18, 19].

On d 0, serial heparinised blood samples were collected at −40, −20, 0, 20, 40, 60, 80, 100, 120, 150, 180, 210, 240, 270, 300, 330, and 390 min relative to the time of ACTH administration to each heifer. Plasma samples were separated by centrifugation at 1600×*g* at 8 °C for 15 min and subsequently stored at −20 °C until assayed.

Cortisol concentration was determined for all heifers at all blood sampling time points, whereas ACTH concentration was determined only at blood sampling time points −40, −20, 20, 80, 120, 180, and 390 min relative to ACTH administration. Plasma cortisol concentrations were determined using the Correlate-EIA kits from Assay Designs (Ann Arbor, MI, USA). The inter- and intra- assay coefficients of variation (CV) were 6.25% and 4.89%, respectively. Plasma ACTH concentrations were measured using a double antibody human ACTH radioimmunoassay (RIA; DiaSorin Inc., Stillwater, MN, USA) previously validated for bovine ACTH [15]. The inter- assay and intra- assay CVs were 8.85% and 7.67%, respectively.

### Hematology

An additional blood sample was collected into a 6-mL evacuated tube containing K_3_EDTA at −40, −20, 0, 20, 80, 150, 270, 330, and 390 min relative to CRH administration for determination of blood hematology variables; white blood cell (WBC) number, red blood cell (RBC) number, hemoglobin (HGB) concentration, hematocrit percentage (HCT %), platelet count (PLT) and total circulating neutrophil, lymphocyte, monocyte, eosinophil, and basophil numbers were determined within 1 h of collection according to the procedures described by Lynch et al. [19].

### Statistical analysis

Data were checked for normality and homogeneity of variance by histograms, QQ-plots, and formal statistical tests as part of the UNIVARIATE procedure of SAS (version 9.1.3; SAS Institute, 2006). Data that were not normally distributed were transformed by raising the variable to the power of lambda. The appropriate lambda value was obtained by conducting a Box-Cox transformation analysis using the TRANSREG procedure of SAS. The transformed data were used to calculate *P*-values. The corresponding least squares means and SE of the non-transformed data are presented in the results for clarity. To evaluate the response over time for plasma concentrations of cortisol, ACTH and haematological variables, these were analysed using repeated measures ANOVA (MIXED procedure), with terms for RFI group, bleed time, pen and their interactions included in the model. Heifer baseline plasma concentrations (mean of −40, −20 and 0 min prior to ACTH administration for cortisol and haematology variables and −40 and −20 for plasma ACTH concentration) were included as covariates. The type of variance-covariance structure used was chosen depending on the magnitude of the Akaike information criterion (AIC) for models run under compound symmetry, unstructured, autoregressive, heterogeneous 1st order autoregressive, or Toeplitz variance-covariance structures. The model with the least AIC value was selected. The differences between mean values for the two RFI groups were determined by *F*-tests using Type III sums of squares. The PDIFF option and the Tukey test were applied as appropriate to evaluate pairwise comparisons between RFI group means. A probability of *P* < 0.05 was selected as the level of significance and statistically tendencies were reported when *P* < 0.10. Spearman correlation coefficients between traits were determined using PROC CORR of SAS.

## Results

### Feed efficiency

Based upon the results of the post-weaning determination of RFI, the low RFI heifers selected for the ACTH challenge had a 19% reduced DMI compared to the high selected RFI animals for the same level of performance. Residual feed intake averaged 0.00 kg DM/d (SD = 0.43) and ranged from −0.87 to 1.02 kg DM/d, equating to a difference of 1.89 kg DM/d between the most and least efficient ranking heifers used in the ACTH challenge . Least squares means for RFI (*P* < 0.001) and F:G (*P* = 0.03; Table 2) were greater for high RFI than for low RFI animals. Heifers in the high RFI and low RFI groups did not differ in final BW (*P* = 0.21) and ADG (*P* = 0.92). Within the group of weanling heifers RFI was positively correlated with DMI (*r* = 0.59; *P* < 0.001). Dry matter intake was positively correlated (*P* < 0.001) with ADG (*r* = 0.55) and negatively correlated (*P* < 0.05) with F:G (*r* = −0.27). Feed conversion ratio was negatively correlated (*P* < 0.001) with ADG during the RFI measurement period (*r* = −0.85).

**Table 2:** Effect of residual feed intake (RFI) ranking on feed intake, efficiency and animal performance in beef heifers

	High RFI	Low RFI	SEM	*P*-value
Number of Animals	12	12	–	–
Residual feed intake, kg/d	0.34	−0.48	0.037	0.001
Feed conversion ratio, kg DM/kg gain	8.13	6.19	0.834	0.03
DMI, kg/d	5.92	4.98	0.127	0.001
Mid-test metabolic BW, kg^0.75^	75	77	1.6	0.27
ADG, kg/d	0.71	0.76	0.028	0.92
Final BW, kg	340	355	9.8	0.21

### Exogenous ACTH challenge

**Fig. 1:**
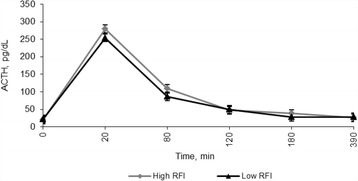
Effect of exogenous adrenocorticotropic (ACTH) administration (1.98 IU/kg metabolic BW^0.75^) on plasma ACTH concentration (pg/dL) in beef heifers differing in phenotypic RFI. The values are expressed as Lsmeans with SEM. RFI, *P* = 0.59; Time, *P* < 0.001; RFI × Time interaction, *P* = 0.13. SEM: High RFI = 4.98; Low RFI = 6.55

**Fig. 2:**
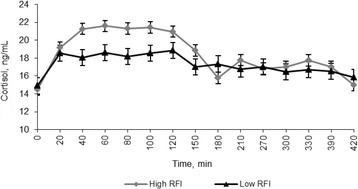
Effect of exogenous adrenocorticotropic (ACTH) administration (1.98 IU/kg metabolic BW^0.75^) on plasma cortisol concentration (ng/mL) in beef heifers differing in phenotypic residual feed intake (RFI). The values are expressed as Lsmeans with SEM. RFI, *P* = 0.45; Time, *P* < 0.001; RFI × Time interaction, *P* = 0.06. SEM: High RFI = 0.712; Low RFI = 0.688

**Fig. 3:**
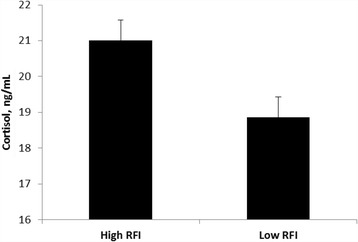
Mean plasma cortisol stress response from 40 min to 150 min relative to exogenous adrenocorticotropic (ACTH) administration (1.98 IU/kg metabolic BW^0.75^) in beef heifers differing in phenotypic residual feed intake (RFI). The values are expressed as Lsmeans with SEM. RFI, *P* = 0.006. SEM: High RFI = 0.57; Low RFI = 0.52

Plasma ACTH and cortisol responses to administration of exogenous ACTH for both RFI groupings are presented Figs. 1 and 2, respectively. Heifers differing in RFI did not differ (*P* = 0.59) in ACTH concentration. Concentration of ACTH peaked (*P* < 0.001) in both RFI groups at 20 min post-ACTH administration, following which concentration declined to baseline levels by 150 min. Similarly, cortisol systemic profile peaked at 60 min and concentrations remained continuously elevated for 150 min. A RFI × time interaction was detected for cortisol concentrations (*P* = 0.06). High RFI heifers had a greater cortisol response than Low RFI from 40 min to 150 min relative to ACTH administration (Fig. 3), whereas there was no difference between RFI grouping subsequently. Cortisol response, from ACTH adrenal stimulation was positively associated with RFI status (*r* = 0.32; *P* < 0.01).

### Haematology

No effect of RFI grouping or a RFI × time interaction (*P >* 0.10; Table 3) was evident for neutrophil, lymphocytes, monocyte, eosinophils and basophil count. Mean plasma RBC number (*P* = 0.02) and HCT percentage (*P* = 0.02) were greater for low RFI than high RFI animals (RBC = 6.07 vs. 6.23; HCT = 23.2 vs. 24.5 for high, and low RFI groups, respectively). An effect of time (*P* < 0.0001; Table 3) was found neutrophil, monocyte, eosinophils and basophil count and for PLT number and HGB with values generally increasing for leucocytes and monocytes, and decreasing for PLT and HGB as time progressed.

**Table 3:** Effect of exogenous adrenocorticotropic (ACTH) administration^a^ on haematology variables in beef heifers differing in phenotypic residual feed intake (RFI)

		Time (min) relative to ACTH administration										
Cell type^b^	RFI Group	0	20	80	150	270	330	390	SEM	RFI	Time	RFI × Time
Neutrophils, × 10^3^ cells/μL	High	9.1	11.7	10.9	11.4	11.9	11.9	12.2	0.36	0.90	0.001	0.92
	Low	8.5	11.3	10.8	12.2	12.9	11.9	11.7	0.41			
Lymphocytes, × 10^3^ cells/μL	High	3.36	3.51	3.3	2.92	3.21	3.41	4.02	0.159	0.99	0.71	0.78
	Low	3.88	3.38	3.16	3.07	3.55	3.32	3.27	0.175			
Monocytes, × 10^3^ cells/μL	High	0.69	0.39	0.53	0.39	0.45	0.47	0.58	0.04	0.99	0.01	0.95
	Low	0.7	0.5	0.68	0.39	0.49	0.38	0.42	0.045			
Eosinophils, × 10^3^ cells/μL	High	0.18	0.08	0.21	0.12	0.12	0.12	0.36	0.027	0.85	0.001	0.06
	Low	0.19	0.21	0.06	0.13	0.19	0.31	0.24	0.031			
Basophils, × 10^3^ cells/μL	High	0.05	0.06	0.05	0.06	0.06	0.06	0.07	0.003	0.61	0.001	0.3
	Low	0.05	0.05	0.05	0.06	0.07	0.06	0.06	0.003			
WBC, × 10^3^ cells/μL	High	16.6	14.7	15.3	14	14.8	14.7	16.3	0.3	0.35	0.53	0.7
	Low	15.4	16.2	16.1	15.3	16.6	15.4	15.3	0.34			
RBC, × 10^6^ cells/μL	High	6.19	6.33	6.04	5.66	6.08	6.03	6.17	0.093	0.02	0.1	0.81
	Low	5.89	6.66	6.42	6.41	6.53	6.43	6.57	0.107			
HGB, g/dL	High	9.46	9.6	9.14	8.63	9.25	9.25	9.54	0.124	0.08	0.04	0.8
	Low	8.77	9.94	9.6	9.51	9.78	9.62	9.81	0.142			
HCT, %	High	23.6	24.3	23.1	21.8	23.3	22.9	23.6	0.36	0.02	0.06	0.87
	Low	22.4	24.5	24.5	24.4	24.8	24.5	25.1	0.42			
MCHC, g/dL	High	40.3	39.5	40	44.8	40.2	40.7	40.9	0.63	0.23	0.71	0.92
	Low	44.3	38.7	38.7	38.8	39.2	39.1	38.8	0.74			
PLT, × 10^3^ cells/μL	High	366.6	312.7	458.9	327	420.5	300.7	346.4	22.82	0.69	0.001	0.76
	Low	309.3	329.4	405.2	269.1	436.3	274.4	310.4	26.11			

^a^Exogenous ACTH (1.98 IU/kg metabolic BW^075^)

^b^ *WBCP* White blood cell number, *RBC* red blood cell number, *HGB* hemoglobin concentration, *HCTH* hematocrit percentage, *MCHC* mean corpuscular hemoglobin concentration, *PLT* platelet number

## Discussion

Understanding the biological mechanisms that control feed efficiency is fundamental to the future of profitable livestock production systems [1, 20]. Susceptibility to stress and the cross regulatory responses between the neuro-endocrine and immune systems have long been postulated as potential processes that contributes to inter- animal variation in feed efficiency in cattle [18]. However, despite the credible link between these traits there is a dearth of published work in this area, which is surprising given the importance of feed efficiency and susceptibility to stress (the implications on health and behaviour) to overall animal production efficiency [2]. Therefore, this study aimed to further enhance our knowledge of the biological control of feed efficiency in cattle by investigating the sensitivity of the HPA axis (a putative regulator of energetic efficiency) to a physiological ACTH stress challenge.

### HPA axis and feed efficiency

Activation of the HPA axis is the main defining feature of the stress response [21] and one of the main biological outcomes in response to a stressor is a rise in metabolic rate [8, 22]. Elevated metabolic rate can influence appetite and energy partitioning and utilisation mainly through altering catabolic process such as increasing the rate of lipolysis and protein degradation [8, 23]. These biological pathways have all been postulated as inherent regulators of an animal’s energetic efficiency [2–5].

To monitor animal’s stress response measuring glucocorticoid hormones and HPA axis activity is the standard approach. Through dynamic testing methodologies the HPA axis can be pharmacologically stimulated with the use of exogenous CRH or ACTH, and response at both the pituitary and adrenal levels can be evaluated. Indeed, HPA axis challenges by way of exogenous CRH or ACTH stimulation have been shown by our group previously [15, 24] and others [25, 26] to be appropriate for investigation of the bovine stress response.

The adrenal cortex was successfully stimulated by ACTH administration in this study and the cortisol response and maximum peak time (60 min) was consistent with previous ACTH challenge experiments [9, 15, 27]. For the ACTH response no difference in plasma concentrations were detected between the divergent RFI phenotypes throughout the physiological challenge, concurring with the results that Adago [28] reported in Braham Cattle. Cortisol is the principal stress biomarker and the biological endpoint for the investigation of HPA function. Knott et al. [29] found that rams selected for greater cortisol concentration following an ACTH challenge also had a higher RFI than their lower cortisol response counterparts. Those authors also attributed up to 35% of the variation in RFI to the changes in cortisol concentrations, indicative of a stress response. Similarly, in a multi-generational selection experiment for RFI in chickens, Luiting and Verstegen [11] reported that inefficient hens had a greater cortisol response following an adrenal ACTH challenge. In cattle, Richardson et al. [30] observed a trend for a positive relationship between blood cortisol concentration of steers and their sire’s estimated breeding value for RFI. Although, admittedly when phenotypic RFI and plasma cortisol concentrations were compared in the Richardson study a negative or inverse relationship was detected between the traits. However, it could also be argued that the ability to truly quantify stress responsiveness or HPA sensitivity in that study may have been somewhat limited, as a physiological stressor/challenge was not imposed on the divergent RFI animals. Results from the current experiment agree with the findings in sheep [6, 29] and chicken [11] indicating that energetic inefficiency is associated with greater susceptibility to stress and is a likely underlying driver along with other biological processes [2] of variation in the trait. Indeed, our work clearly shows a greater cortisol stress response in High RFI heifers following activation of the HPA axis with exogenous ACTH (adrenal stimulation). In contrast to these findings Kelly et al. [18], failed to show an association between feed efficiency and stress biomarkers when focusing on neuroendocrine responses to a physiological CRH stress challenge. In that study HPA axis stimulation was via exogenous CRH and a more diminished adrenal cortex response was observed relative to that measured in the present study, which may explain the disagreement in results between our study and that of Kelly et al. [18]. Indeed, in all other published feed efficiency studies that investigated physiological stressors [6, 11], ACTH which was the sectretagogue used to examine adrenal stimulation of HPA and subsequent stress response. Foote et al. [31] also showed in cattle that efficient or low RFI heifers have lower concentrations of fecal corticosterone than the high RFI heifers and fecal corticosterone was identified as a useful physiological indicator for feed efficiency in finishing beef cattle. This finding complements our results, in that corticosterone is an informative representative of adrenal activity and is a glucocorticoid produced in a pattern similar to cortisol in response to ACTH release.

#### Immune response and feed efficiency

The central nervous system regulation of the immune system acts principally via the HPA [32, 33]. Neutrophils, as well as many other immune cells, are well known targets of stress hormones, possessing receptors for catecholamines and glucocorticoids secreted during an acute stress response. A profound leukocytosis, with marked neutrophilia, has often been observed in association with elevated circulating glucocorticoids [8, 23] indicating a disruption of neutrophil homeostasis in response to stress in cattle. This cross regulation between neuro-endocrine-immune systems is critical for homeostasis and has profound effects on the health, behaviour and performance of animals [33]. In general, the data from the current trial failed to show a relationship between feed efficiency, and the principal indicators of immune-competence in response to a physiological stressor. When Kelly et al. [18] investigated neuroendocrine responses to an exogenous physiological CRH physiological challenge in animals of differing RFI status, they did not find an association between haematological subpopulations and RFI. Additionally, Lawrence et al. [13] reported that high and low RFI heifers did not differ in any of the haematology variables measured, except at parturition when lymphocyte and monocyte counts were positively associated with RFI. Theis et al. [34] did not detect any relationship between phenotypic RFI with a similar set of haematological variables as was reported on in this study. However, recent work from our group [35] has shown that feed efficient pigs had lower gene expression profiles for intestinal innate immune response genes following a lipopolysaccharide (LPS) challenge compared to their inefficient contemporaries, suggesting that feed efficient animals have an altered immune response to infection. Indeed, mounting effective innate immune responses is metabolically costly to the animal which demands a nutrient tradeoff at the expense of other biological demanding processes and as such could be an underlying contributor to variation in energetic efficiency [36]. Richardson et al. [37] reported a genetic association between sire estimated breeding value (EBV) for RFI with white blood cell count, lymphocyte count and haemoglobin level, in crossbred beef steers. Gomes et al. [38] also reported that white blood cell count was lower and haemoglobin concentration was higher in high RFI heifers than low RFI heifers. In our study, we did detect that red blood cell count and hematocrit percentage was greater in Low RFI compared to High RFI animals, potentially indicating some deviations in oxygen requirement or carbon dioxide transport within their haem structure due to stress sensitivity [39].

## Conclusions

The data presented provide evidence that animals differing in feed efficiency have an altered HPA axis function in response to a physiological stressor (ACTH adrenal stimuli) and susceptibility to stress and responsiveness of the HPA axis is likely to contribute to appreciable variation in the efficiency feed utilisation of cattle. As such, these findings add greater clarity and focus to the body of published work examining the biological control of feed efficiency in cattle.

## Abbreviations

ACTH: Adrenocorticotropic hormone
ADG: Average daily gain
BW: Bodyweight
CRH: Corticotrophin-releasing hormone
CV: Co-efficient of variation
DMI: Dry matter intake
EBV: Estimated breeding value
F:G: Feed conversion ratio
HCT %: Hematocrit percentage
HGB: Hemoglobin
HPA: Hypothalamic-pituitary-adrenal
LPS: Lipopolysaccharide
MBW: Metabolic body weight
RBC: Red blood cell
RFI: Residual feed intake
WBC: white blood cell
